# Continuous *in vivo* blood pressure measurements using a fully implantable wireless SAW sensor

**DOI:** 10.1007/s10544-013-9759-7

**Published:** 2013-04-05

**Authors:** Olive H. Murphy, Mohammad Reza Bahmanyar, Alessandro Borghi, Christopher N. McLeod, Manoraj Navaratnarajah, Magdi H. Yacoub, Christofer Toumazou

**Affiliations:** 1 Centre for Bio-Inspired Technology, Institute of Biomedical Engineering and the Department of Electrical and Electronic Engineering, Imperial College London, London, SW7 2AZ UK; 2 The Heart Science Centre, Harefield Hospital, Hill End Road, Harefield, Middlesex, UB9 6JH UK

**Keywords:** SAW, Pressure sensor, Implantable, Patient monitoring, Telemetry, Telemedicine, Heart, Wireless

## Abstract

In this paper, the development of a fully implantable wireless sensor able to provide continuous real-time accurate pressure measurements is presented. Surface Acoustic Wave (SAW) technology was used to deposit resonators on crystalline quartz wafers; the wafers were then assembled to produce a pressure sensitive device. Excitation and reading via a miniature antenna attached to the pressure sensor enables continuous external interrogation. The main advantages of such a configuration are the long term stability of quartz and the low power necessary for the interrogation, which allows 24/7 interrogation by means of a hand-held, battery powered device. Such data are of vital importance to clinicians monitoring and treating the effects of hypertension and heart failure. A prototype was designed and tested using both a bio-phantom test rig and an animal model. The pressure traces for both compare very well with a commercially available catheter tip pressure transducer. The work presented in this paper is the first known wireless pressure data from the left ventricle of the heart of a living swine.

## Introduction

Correct blood pressure management is key in the treatment of a number of conditions, such as hypertension and heart failure (Gradman 2012; Hoeper and Granton 2011). Ambulatory blood pressure measurements (i.e. un-tethered 24 h measurement by means of an automated system) have been shown to have higher reproducibility compared to office measurements (i.e. when taken at the doctor’s surgery) due to the so called “white coat hypertension” phenomenon (Mansoor et al. 1994). Furthermore, ambulatory measurements provide information about all of the patient’s activities, including sleeping and awake modes, which would be otherwise unavailable (White 2006). Chronic monitoring of blood pressure in ambulatory patients also provides useful telehealth and alarm facilities (McManus et al. 2008); however, current telehealth systems are based on intermittent spot measurements with limited accuracy and may therefore be unsuitable for some applications where accuracy is essential (i.e. hypertension where classification steps are in the range of 10 mmHg) (Green et al. 2008). Furthermore, due to the low sampling frequency of these devices (typically 2 to 12 times a day) the development of critical vital signs between measurements could be missed, leading to inaccurate diagnosis/monitoring.

A number of studies have been performed to assess the feasibility and usefulness of continuous hemodynamic monitoring. As early as 2004 Najafi and Ludomirsky (2004) reported on very promising measurements taken in the aorta of live canine models using a microelectromechanical system (MEMS) based pressure sensor and magnetic telemetery; however, no reported human trials followed. Rozenman et al. (2007) implanted a miniature device in animals as well as in a patient cohort; the sensor was based on an acoustically powered piezoelectric transducer with a custom-built, low-power control chip able to transmit pressure measurements continuously for 5–10 s. Readings were simultaneously taken with a catheter tip transducer advanced to the same location and good agreement was found, with a maximum deviation of less than 5 mmHg. A similar protocol was used by Verdejo et al. (2007) to evaluate the accuracy of the CardioMEMS^TM^ heart failure sensor also based on a (MEMS) pressure-sensitive capacitor. The sensor was electromagnetically coupled to an external antenna, which powers the devices and subsequently captures its resonant frequency, which is related to the arterial pressure. The method was validated by comparison with currently available methods and proved later in a blind trial its usefulness in improving treatment and decreasing the rate of re-hospitalization (Abraham et al. 2011). A different system was used by Ritzema et al. (2007) to measure left atrial pressure (LAP) in ambulatory heart failure patients: an implantable sensor coupled with a subcutaneous antenna coil was implanted by means of transseptal puncture and subsequently interrogated by means of radio frequency (RF) excitation transmitted by an external module. The interrogation was performed by the patients at prescribed times during the day and the data was then transmitted for offline analysis. Similar systems have also been developed and tested for measuring pressure in other organs (Chitnis et al. 2012; Tan et al. 2009). An alternative to intra-arterial pressure measurements is the use of extra-arterial blood pressure monitors; however, it has been determined that their performance is not yet good enough to achieve clinical success (Potkay 2008).

This paper presents a wireless, ambulatory, continuous blood pressure monitoring system which is able to provide real-time accurate pressure measurements. The location of the implant is the left ventricle (LV), the pressure here being vital for monitoring post heart transplant patients and patients with left-ventricle assist devices (LVAD). With regards to the latter, palpable pulses are not always present so traditional cuff measurements are difficult and while other non-invasive methods using doppler are advised, the associated difficulties are known and have been widely published (Slaughter et al. 2010). Future developments will lead to less invasive implantation procedures, any disadvantages of which will be overcome by the benefits of 24/7 local pressure measurements. This information is highly desirable by clinicians and, furthermore, close pressure monitoring has proved useful in providing directions regarding treatment and discharge of patients undergoing LVAD implantation (Wieselthaler et al. 2001).

A system overview will be presented in Section 2 with more detail given for the sensor, antenna, assembly and interrogator presented in the following sections. *In vitro* testing was carried out before *in vivo* experiments were performed on a swine animal model as seen in Sections 7.1 and 7.2, respectively. Wireless pressure readings show very good comparison to a commercially available catheter tip pressure transducer.

## System overview

**Fig. 1:**
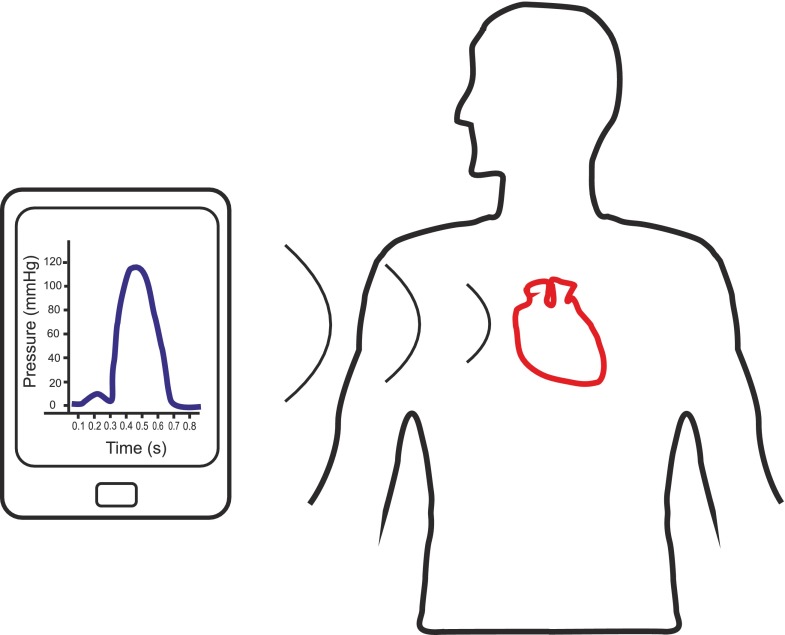
System overview

The system comprises implantable components and external components. Figure 1 presents an overview of the system, whereby an external interrogator powers the implantable passive (batteryless) sensor using electromagnetic radiation; it then listens for the response and interprets an accurate pressure reading. In this paper, the external component is a larger interrogation system with the potential to optimize and reduce its size in future iterations.

**Fig. 2:**
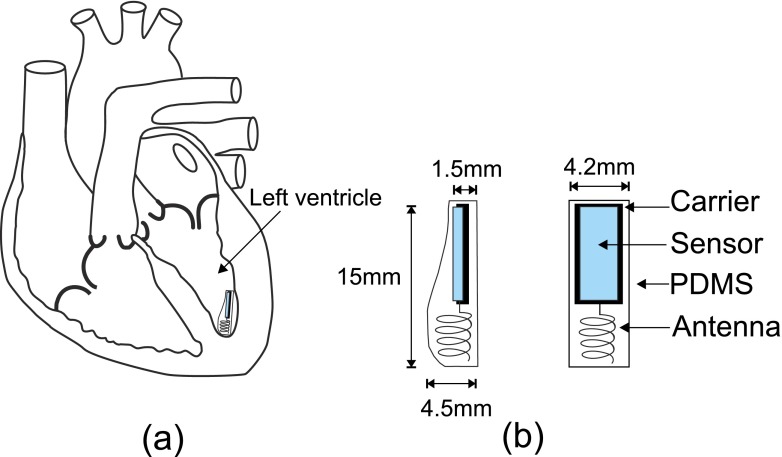
**a** Location of the pressure sensor in the *left ventricle*, **b** dimensions of the implantable sensor

Figure 2a shows the exact location of the implantable sensor in the left ventricle of the heart. Figure 2b shows the sensor, carrier and antenna all within bio-compatible polydimethylsiloxane (PDMS) along with the dimensions of same.

A more detailed explanation will be given in the following sections.

## Pressure sensor

### Surface acoustic wave resonator

Wave propagation along the surface of an elastic solid was first discussed by Lord Rayleigh in 1885 (Rayleigh 1885) but engineering interest in this phenomenon was essentially triggered in 1965 when the use of interdigital electrodes for surface acoustic wave excitation on a piezoelectric substrate was first reported (White and Voltmer 1965). Soon after this invention, SAW devices were recognized by the electronics industry and a variety of devices, including resonators, entered the commercial domain. A number of different piezoelectric materials may be used as a substrate to produce SAW resonators, each having their advantages and drawbacks. Electromechanical coupling factor, temperature and long term stability are among the parameters that determine the choice of the substrate for a particular application (Spassov et al. 2008). Single crystal quartz wafers of high quality are commercially available at reasonable cost; moreover, certain cuts of quartz may be chosen to produce high quality factor resonators whose natural frequencies are insensitive to temperature variations around a desired operating point (Hashimoto 2000; Pohl 2000). These qualities make quartz SAW resonator a suitable candidate for long term implantable sensors.

SAW resonators are produced by depositing metallic interdigital transducer (IDT) electrodes together with reflecting gratings on a piezoelectric substrate. Many parameters define the resonant frequency of a SAW resonator but the dependence of resonant frequency on SAW wave propagation velocity is particularly of interest in pressure sensors. It has been shown that this propagation velocity is dependent on stresses that are induced in the substrate (Cullen and Reeder 1975; Bigler et al. 1989). The subsequent effect on the strain has been used to construct sensors to measure different mechanical quantities including pressure (Seifert et al. 1994; Pohl and Steindl 1998; Das et al. 1976; Vlassov et al. 1993; Risch 1984; Benetti et al. 2008) and has been employed in this research, the basic principle of which will be outlined in the following section.

### Sensor structure and principle of operation

**Fig. 3:**
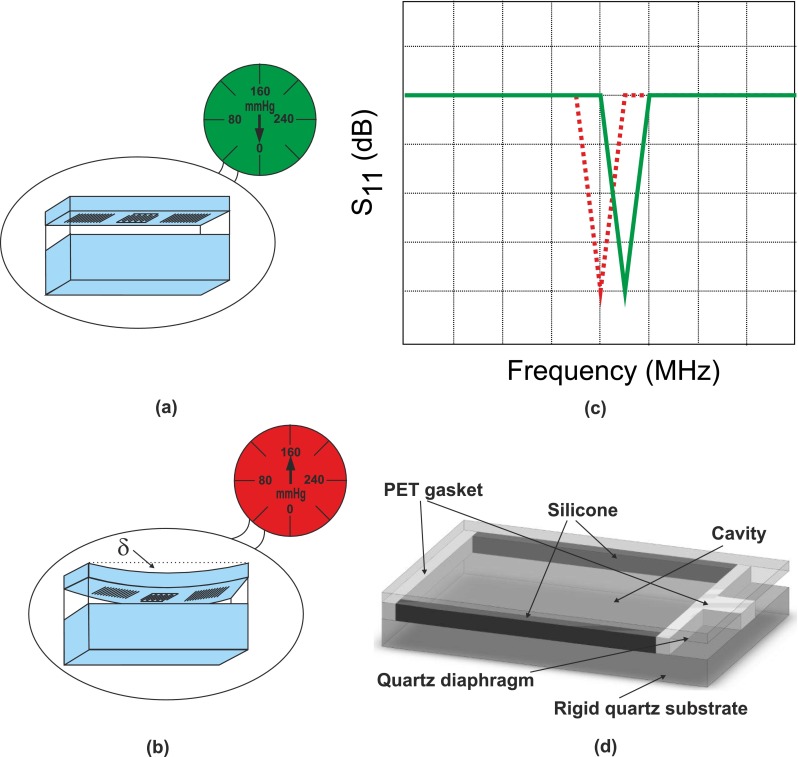
Diagram of the fundamental operation of a SAW pressure sensor with **a** no applied pressure; **b** applied pressure and the corresponding deflection (*δ*) and **c** the frequency response of the SAW pressure sensor with no applied pressure (*green/solid*) and with applied pressure (*red/dotted*). **d** 3D representation of pressure sensor used in this work

Figure 3 shows the principle of operation of a SAW pressure sensor. A thin quartz membrane with a SAW resonator at its centre is placed on top of a cavity (in air) and bonded around its perimeter to form a sealed cavity with a thicker bottom substrate. When no pressure is applied, the membrane does not deflect as shown in Fig. 3a and the resonant frequency $f_0$ is given by the green/solid line in Fig. 3c. Upon increasing the pressure (above 1 atm), the quartz membrane deflects into the cavity as seen in Fig. 3b. This resultant strain in the quartz membrane causes the resonant frequency of the SAW resonator to change as shown in Fig. 3c (red/dotted line). According to Seifert et al. (1994)

1 $$\epsilon = \frac{\Delta P}{P} = -\frac{\Delta f_0}{f_0}$$

where 𝜖 is the induced strain and *P* is the pitch between the IDTs. This frequency shift can be detected and interpreted as applied pressure. It is worth mentioning that the pressure measured by this sensor is relative to 1 atm and therefore measurements should be corrected according the environmental pressure.

Figure 3d shows a 3D representation of the pressure sensor used in this work, where the top plate is supported by two opposing rigid walls (polyethylene terephthalate—PET) and two opposing soft walls (silicone). This configuration allows for larger strains in the centre of the top plate and enhances the sensitivity of the sensor.

### Mechanical characterization

**Fig. 4:**
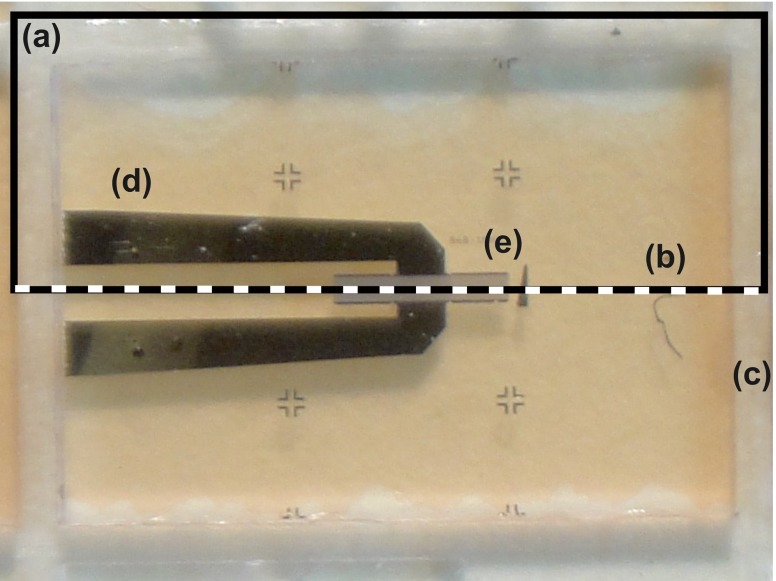
Top view of the SAW resonator: **a** the *rectangle* highlights the area which is reproduced in the FEM model; **b** the *dotted line* showing the axis of symmetry; **c** the *dice lines*; **d** the metal tracks and **e** the SAW resonator

**Fig. 5:**
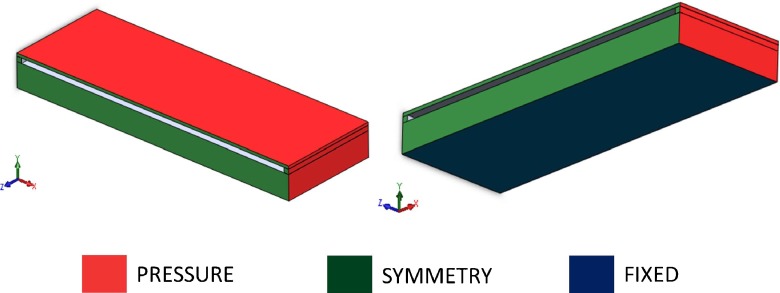
3D visualization of the sensor finite element model. *Colours* relate to faces where loads (pressure) and boundary conditions (symmetry, fixed) are applied

A sensor model was created by means of a commercial CAD package (Solidworks®). The sensor was modelled as a 5.3 by 4 mm top plate having a thickness of 60 μm suspended and connected on the four sides to a bottom plate having the same dimensions but with a thickness of 500 μm. The two plates were connected on the four sides by walls having a rectangular cross-section with a 100 by 250 μm side. The model was subsequently cut in half along the longitudinal axis of symmetry and only half was modelled (see Figs. 4 and 5). Finite element modelling (FEM) was used to simulate the effect of left ventricular pressure on the sensor and for calculating stresses and strains. The top and bottom plate were modelled as crystalline quartz, having Young’s modulus *E* =  76 GPa and poisson’s ratio *ν* = 0.14 (Buff et al. 1998). The two short walls were assumed to have the same material properties as the quartz (rigid walls) while the long wall was modelled as silicone (soft wall) having *E* = 2 MPa and *ν* = 0.49 (Schneider et al. 2008). The structure was subject to 26.6 kPa pressure (equivalent to 200 mmHg blood pressure, see Fig. 5) on all the faces. Boundary conditions were imposed on the bottom of the bottom plate (fixed translation and rotation) and symmetry plane (symmetry condition, Fig. 5). Deflection and strain in the symmetry axis were analyzed. The effect of the PDMS encapsulation was analysed by modelling the PDMS coating. PDMS was modelled as an isotropic material having *E* = 750 kPa and *ν* = 0.49 (Lotters et al. 1997).

**Fig. 6:**
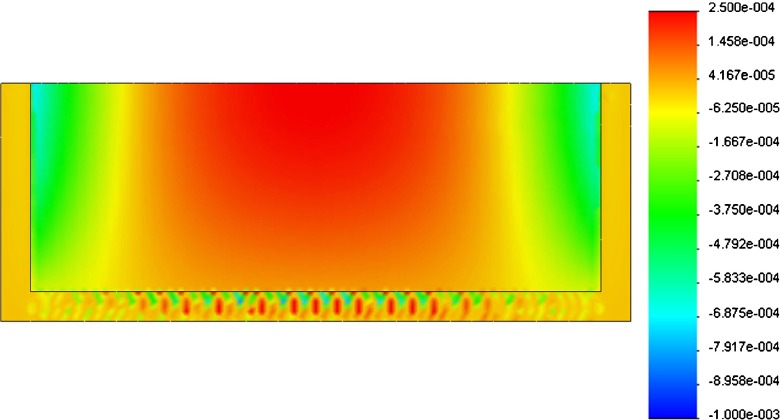
Strain pattern (*ε*) on the bottom surface of the top plate. Positive strain is in *red* while negative strain is in *blue*

Figure 6 shows the strain pattern acting on the bottom surface of the top plate, where the SAW sensor is deposited. The peak deflection ($\delta _{\max }$) was compared with the relative analytical results for a beam subject to uniform load (blood pressure in this case):

2 $$\delta_{\max}=\alpha \frac{p L^4}{E I}$$

where *L* is the length (5.3 mm in our case), *p* is the pressure (200 mmHg), *E* is the young’s modulus of the quartz, *I* is the second moment of area and *α* is a constant whose value depends on the boundary conditions; it is equal to 1/384 in case of fully supported beam (both deflections and rotations are fixed at the two boundaries) and equal to 5/384 in case of simply supported beam (boundary deflections are fixed while rotations are free) (Roarck and Young 1988).

Table 1 shows the values of peak deflection ($\delta _{\max }$), stress ($\sigma _{\max }$) and strain ($\varepsilon _{\max }$) in the three cases: simulated FEM solution and calculated simply supported and fully supported beams.

**Table 1:** Peak deflection ($\delta _{\max }$), stress ($\sigma _{\max }$) and strain($\varepsilon _{\max }$) values for the simulated FEM model compared to calculated solutions in case of fully supported and simply supported beam

	$\delta _{\max}$ (μm)	$\sigma_{\max }$ (MPa)	$\varepsilon _{\max }$ (mm/mm)
FEM	14.8	22.3	2.56E-04
Fully supported	40.05	104	1.37E-03
Simply supported	200.27	156	2.05E-03

**Fig. 7:**
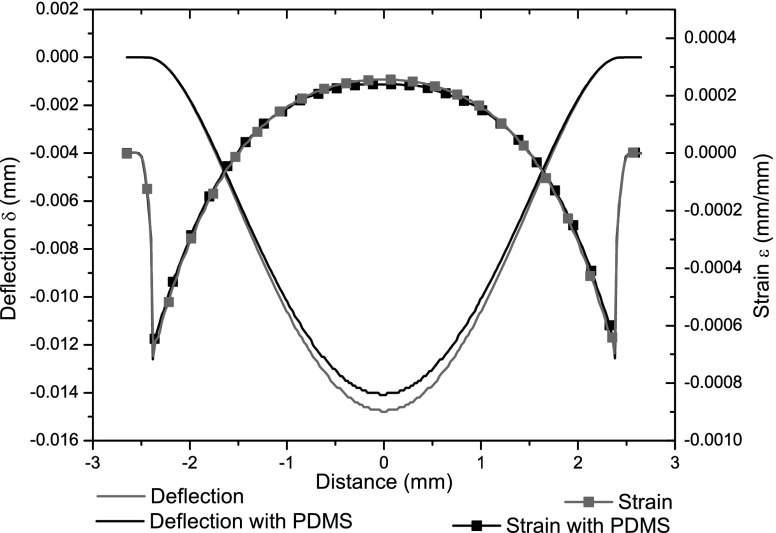
Mid-axis strain and deflection curves for the sensor model. The presence of PDMS (*black curves*) causes a decrease in strain and deflection, compared to the basic model (*grey curves*)

The effect of insulation around the device was assessed by modeling the PDMS encapsulation: Fig 7 shows the change in symmetry axis strain and deflection values. Peak deflection decreases by 4.75 % while peak strain drops by 6.24 %.

### Sensor assembly

#### Preparation of quartz diaphragm

**Fig. 8:**
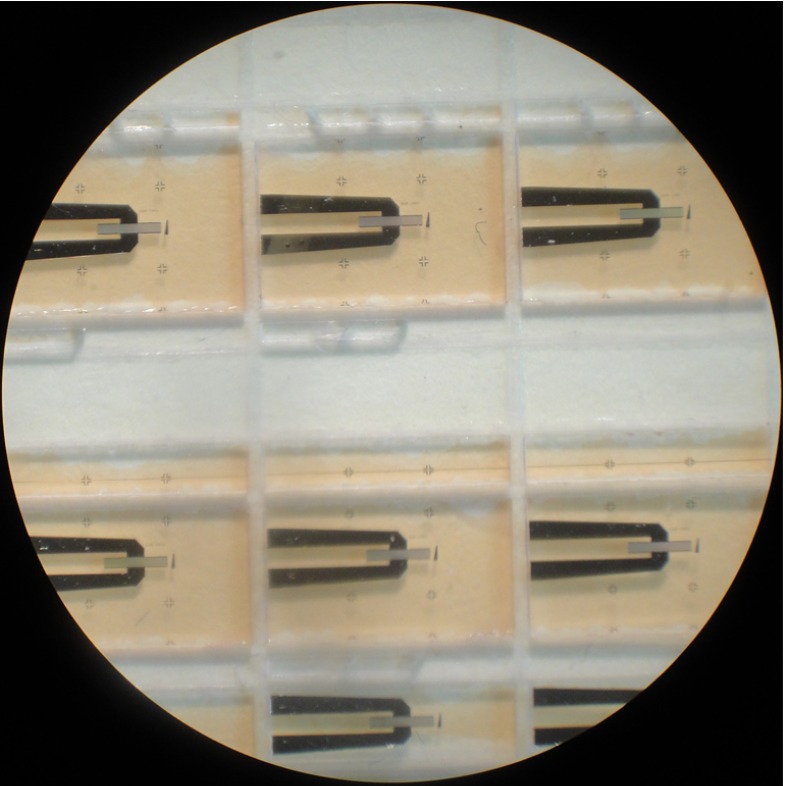
Diced resonators coated with photoresist

**Fig. 9:**
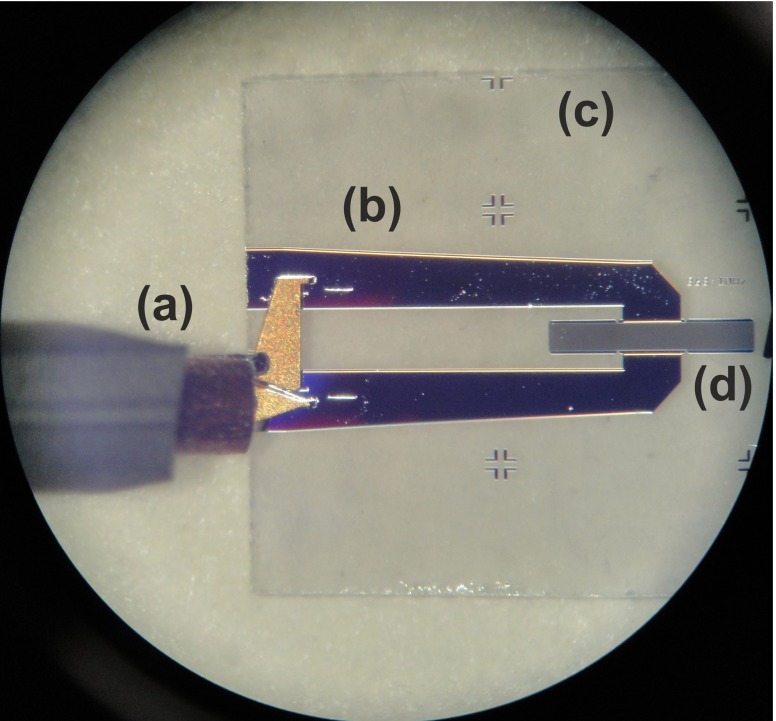
Probing the thin quartz resonator: **a** the RF probe; **b** the metal tracks; **c** the quartz substrate and **d** the SAW resonator

The top plate of the pressure sensor is a 6 by 4 mm thin quartz diaphragm on which a SAW resonator is deposited. The aluminium resonators were deposited on 500 μm thick 100 mm quartz wafer. In order to produce such elements, the thick quartz wafer should be diced and thinned. Since SAW resonators are extremely sensitive to mass loading, any contamination due to mechanical processing and handling should be avoided. Mechanical handling may also damage the delicate metallization and cause device failure. Therefore, a protective layer is required prior to any further processing. To protect the resonators, a thin layer of positive tone phororesist AZ4533 was spin coated, on the metallization side, at 4000 rpm for 5 s and baked at 75 °C for 2 h. Figure 8 shows diced quartz resonators coated with the protective layer. The reason for choosing a positive photoresist is to prevent cross linking due to subsequent exposure to light during thinning and handling. The wafer was then diced using a 250 μm thick dicing wheel and the diced pieces were thinned by mechanical polishing. The quartz diaphragms produced in this way were washed in acetone, isopropyl alcohol (IPA) and deionized water to remove the protective layer. To ensure that no damage had occurred during the thinning process, the resonators were probed after washing, as shown in Fig. 9.

#### Substrate preparation

**Fig. 10:**
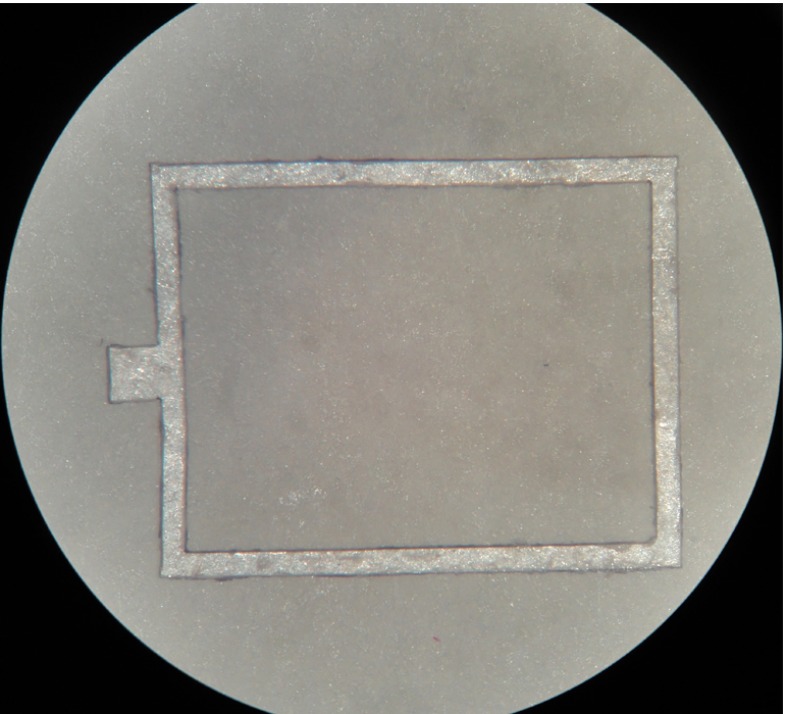
Laser cut gasket

To form a sealed cavity, a 500 μm thick quartz wafer was diced to produce a substrate of the same dimensions as the thin diaphragm. Rectangular gaskets were laser cut from a PET sheet and used as spacers between the quartz substrate and thinned diaphragm (Fig. 10). The gaskets were glued on both surfaces using epoxy to form a sealed cavity of external dimensions 5.3 by 4 by 0.66 mm and internal dimension 4.8 by 3.5 by 0.1 mm. To increase the sensor’s sensitivity, the two long sides of the gasket were removed and the cavity was sealed by silicone as illustrated in Fig. 3.

#### Sensor characterization and testing

**Fig. 11:**
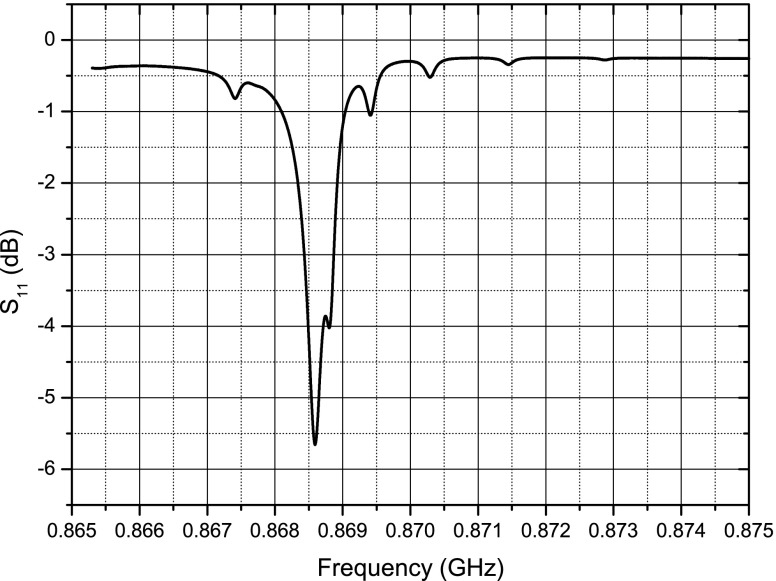
Thin quartz resonator frequency response

A number of thinned quartz membranes were probed and one with the desired response was chosen to form the sensor. The criteria in choosing a membrane were the resonator’s centre frequency, impedance and quality factor. The resonant frequency and impedance are particularly important to ensure that the best possible matching with the antenna is achieved. Also, resonators with higher quality factors were chosen to increase the duration of the response signal. Wafer level probing data showed a tolerance of approximately 150 kHz for the resonant frequency of the resonators. The loaded quality factor of the resonators was in the range of 3100–3800, approximately. Figure 11 shows the plot of ${S_{11}}$ versus frequency for the SAW resonator on the thinned quartz membrane which was used for the sensor assembly.

**Fig. 12:**
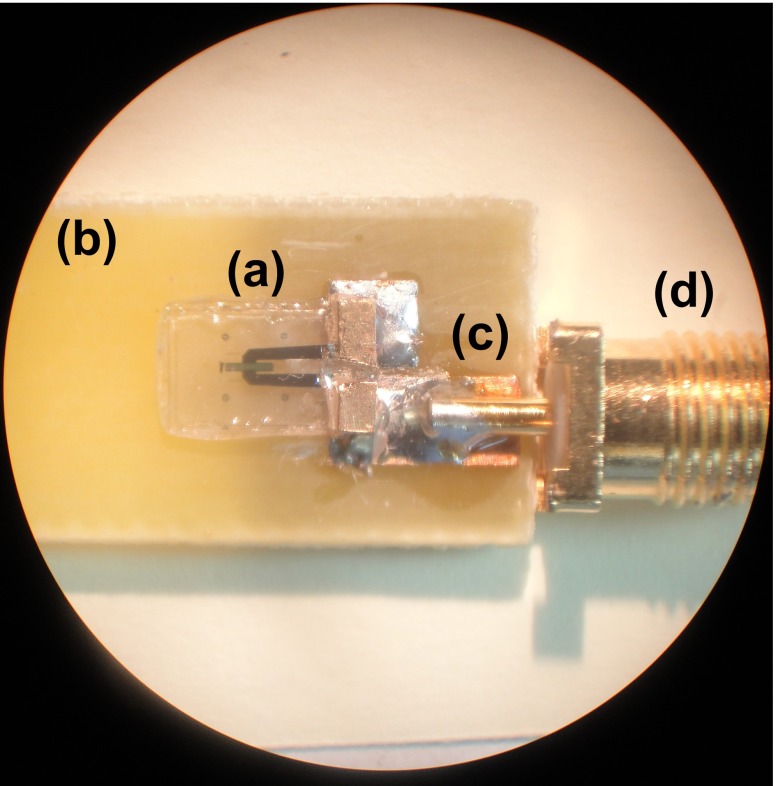
Assembled **a** pressure sensor on **b** FR-4 test board with **c** transmission line and **d** SMA connector

**Fig. 13:**
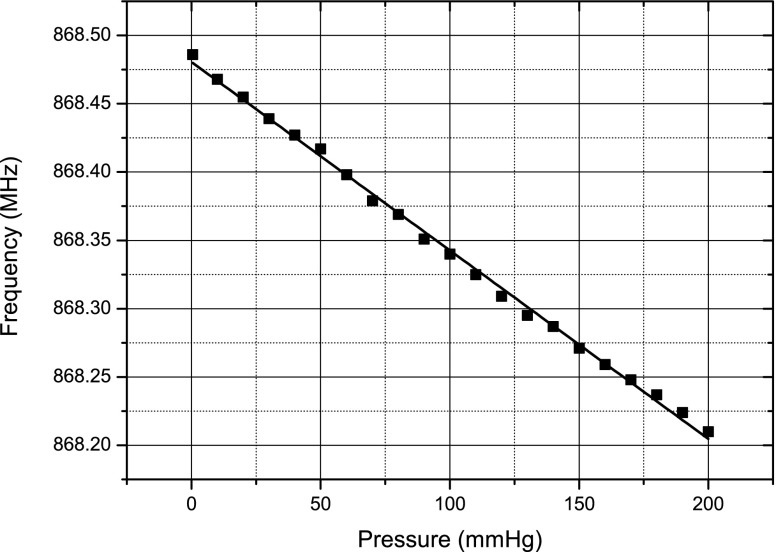
Plot of the sensor resonant frequency versus pressure

After assembly, the sensor was connected to a Rhode and Schwarz® ZVL Vector Network Analyzer via a FR-4 test board (Fig. 12) and placed inside a pressure chamber. To test for any gross leaks from the cavity, the chamber pressure was increased and held at 100 mmHg for 5 min. The resonant frequency of the sensor was observed for any shifts to ensure that the cavity remained sealed. The chamber was then pumped with air and the pressure was increased from 0 to 200 mmHg, in steps of 10 mmHg, the frequencies at which ${S_{11}}$ were minimum were tracked and recorded. It should be pointed out that no measurements were performed in the reverse direction to observe hysteresis in the sensor. Figure 13 shows a plot of these frequencies against pressure.

## Implantable antenna

**Fig. 14:**
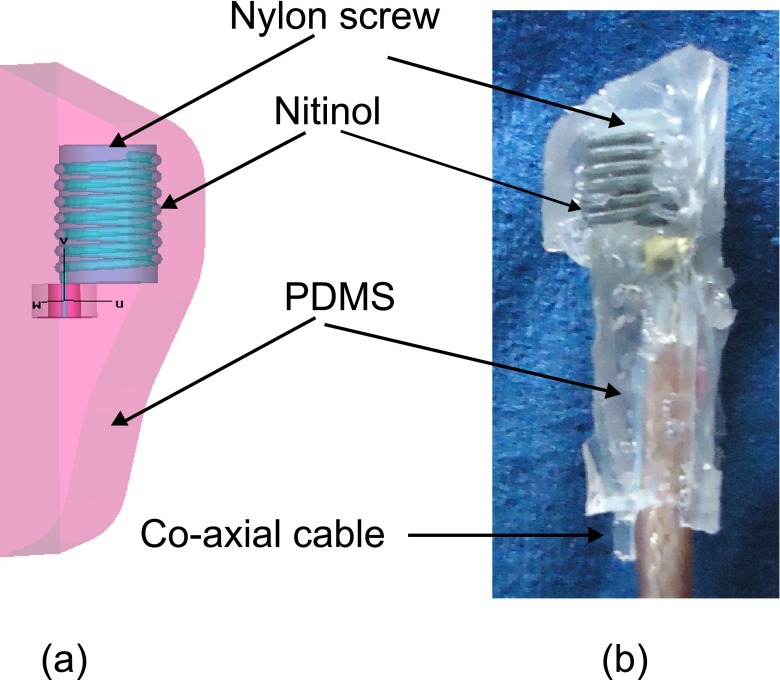
**a** *Pseudo*-normal-mode helical antenna wound on a nylon screw, surrounded by PDMS and simulated in a homogenous human body environment (**b**) photo of the antenna used for tuning in bio-phantom

The design, simulation, *in vitro* and *in vivo* verification of the antenna used here has been described in detail elsewhere (Murphy et al. 2012). This implantable antenna is designed for use in the left ventricle, taking into account the total space that is available for the implant. A *pseudo* normal mode helical antenna provides circular polarization and is therefore suitable for placement in the body when the exact orientation of the implant is difficult to ascertain after implantation. This antenna geometry does add to the overall height of the implant as can be seen in Figs. 2b and 14a and b, but other reported antennas suitable for implantation (Kim and Rahmat-Samii 2004; Soontornpipit et al. 2004; Merli et al. 2011; Occhiuzzi et al. 2012) are not suitable for use in the left ventricle.

**Fig. 15:**
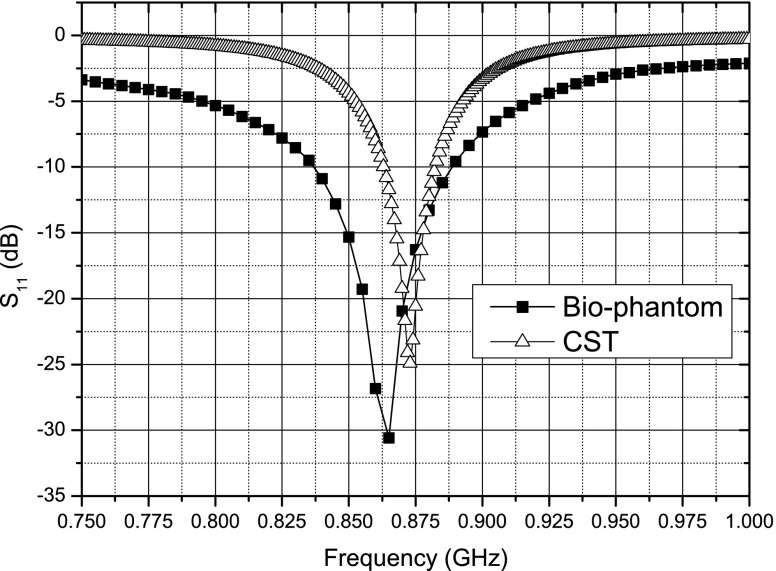
Simulated (CST) and *in vitro* (Bio-phantom) frequency response of a *pseudo*-normal-mode helical antenna for use in the left ventricle

In comparison to Murphy et al. (2012) the geometry of the insulation differs as the sensor is now included and was designed to minimize blood flow disturbance. The insulation is made from PDMS and the antenna was wound on a nylon screw for support as seen in Fig 14a. The original design was retuned in CST Microwave Studio^TM^ to account for any frequency detuning due to the extra insulation. Figure 15 shows the tuned response of the antenna ($S_{11}$-CST).

As per Murphy et al. (2012) the antenna is made and attached to a co-axial cable (see Fig. 14b) and tested in electromagnetically correct bio-phantom to mimic the human body. In this case no further retuning was required as can be seen in Fig. 15 ($S_{11}$ Bio-phantom)). Also, as was seen (Murphy et al. 2012), the higher resistive losses of the Nitinol show a more broadband response than that predicted by the simulations, but this will not have any impact on this application.

## Implant assembly

The helical antenna was connected to one of the resonator pads using conductive epoxy CW2400 from Chemtronics®. The other resonator pad was connected in the same way to a copper tape attached under the substrate acting as a ground plane. The produced assembly was placed into a rapid prototyped mould and cast in PDMS to protect the sensor and insulate the antenna. In order to remove any bubbles from the PDMS insulation, the assembled sensor and antenna was placed in a degassing chamber.

## The interrogator system

The natural frequency of the SAW resonator is the quantity of interest that should be measured wirelessly. To this end, a pulsed RF signal is generated and used to excite the SAW resonator. The RF frequency of the pulse is around 868 MHz (close to the resonant frequency of the SAW) and the pulse repetition frequency (PRF) is set to 100 Hz. The system was designed to have an adjustable pulse width so that the minimum required pulse width for efficient excitation could be determined. The radiated RF pulse energy should be transferred to the resonator as efficiently as possible, therefore as mentioned above, the resonator is connected to an antenna which is tuned around its resonant frequency. After a short period of time (∼1 μs), the resonator reaches its steady state and the RF excitation is turned off. The resonator energy will then be radiated back through the implantable antenna as a decaying sine wave at the SAW resonant frequency. This is typically a decaying signal of duration of 1–2 μs and is the signal of interest. Recording of this signal starts with a short delay of around 250 ns after the RF excitation is switched off. This delay time is introduced in order to avoid recording any reflections of the excitation pulse from surrounding objects.

**Fig. 16:**
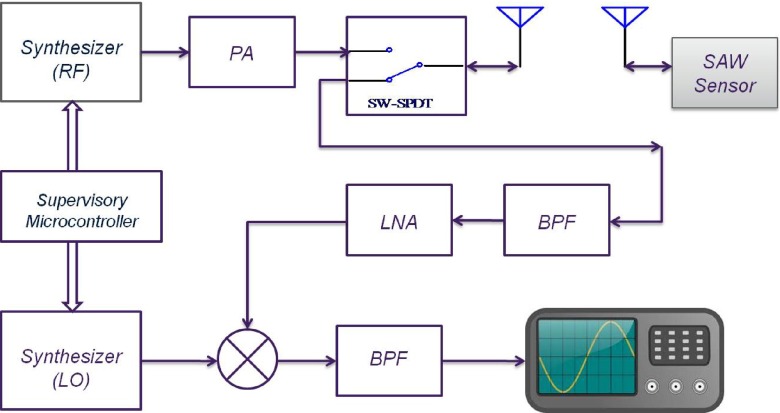
Simplified block diagram of the interrogating system

The simplified block diagram of the interrogating system is shown in Fig. 16. The system consists of two programmable synthesizers to produce the RF and mixing signals. In order to obtain the maximum signal to noise ratio for different resonators, both frequency and amplitude of these signals can be varied by the user. The centre frequencies of individual resonators have a tolerance range of 100 kHz and therefore excitation frequency should be adjustable to ensure that any resonator is excited close to its resonant frequency. At the beginning of each interrogation, the system is switched to transmit-mode sending an RF pulse of adjustable width to excite the resonator. The pulse is then turned off and after a delay time of 250 ns, the system is switched to its receiving mode. In this mode the system is essentially functioning as a super heterodyne receiver consisting of band pass image rejection filters, a chain of low noise amplifiers (LNAs) and a mixer by which the received signal is down converted to an intermediate frequency (IF) at 50 MHz. This signal is recorded by an Agilent oscilloscope model DSO5012A that is capable of signal acquisition in segmented memory mode. This mode of data acquisition is particularly helpful in pulsed RF applications where short bursts of high frequency signals, that are required to be sampled with a high sampling rate, occur at a much lower frequency. In this case, for example, the RF bursts were downconverted to IF and sampled at 1 GSPS. The segmented memory mode avoids recording the dead signal between the bursts.

**Fig. 17:**
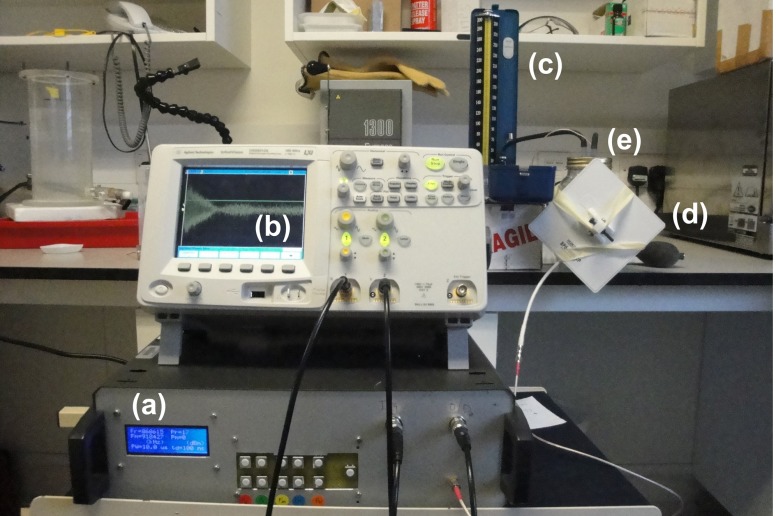
Interrogating the implant placed in biophantom: **a** the RF interrogator; **b** the Agilent oscilloscope; **c** a standard sphygmomanometer; **d** a patch antenna and **e** the pressure chamber containing the bio-phantom and pressure sensor

Figure 17 shows the interrogating system as well as the pressure chamber that was used as a test rig to interrogate the pressure sensors. The data acquired from the RF bursts were processed and a frequency sample point was estimated for each burst using an algorithm based on chirp z-transform (Rabiner et al. 1969).

## Experimental procedures

### *In vitro* measurements

To test the sensor, a testing rig able to simulate LV pressure conditions was created. A heart pump simulator (Placepower, UK LTD, Norfolk, UK), comprising a stepper motor that drives a reciprocating ball screw slide, ending with a piston, was connected to a hermetically sealed water reservoir. The water reservoir had an outlet on the top, connected to a second cylinder containing bio-phantom. This configuration allowed pressure transfer between the reservoir and the cylinder without contact between the water and bio-phantom. The sensor was inserted into the bio-phantom and a commercially available catheter-tip transducer (Mikro-Tip Pressure Catheter, Millar Instruments, Texas) was inserted into the cylinder. The catheter-tip transducer was connected to a data acquisition system (MP36R, Biopac Systems Inc., Goleta, CA). A 50 Hz low-pass filter was used during the recording. The heart pump simulator was actioned and pressure levels in the cylinder were simultaneously recorded from the device and from the catheter-tip transducer.

**Fig. 18:**
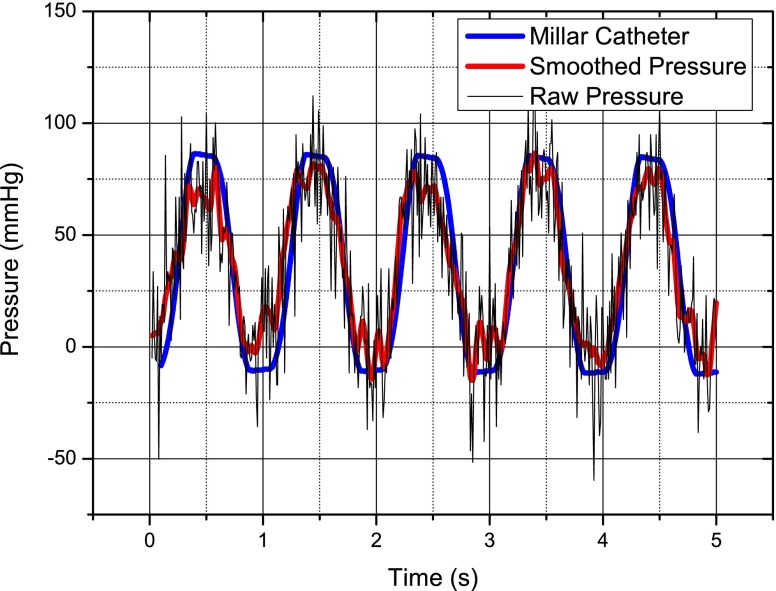
Simulated left ventricle pressure response versus time for the wireless pressure sensor and a catheter-tip transducer—it may be noted that the pressure does not return to zero but goes negative—this is due to slight leaks in the heart pump system

The sensor is activated using a patch antenna placed close to the bio-phantom and attached to the interrogator and the received signal was recorded, also using the interrogator and a high speed digital scope. For the purpose of calibration, a baseline pressure is recorded when the pump is off.The pressure versus time is shown in Fig. 18, there is significant noise on the raw data; however, some simple smoothing in Matlab® cleans this significantly and the sensor compares very favorably with the catheter tip pressure transducer.

### *In vivo* measurements

**Fig. 19:**
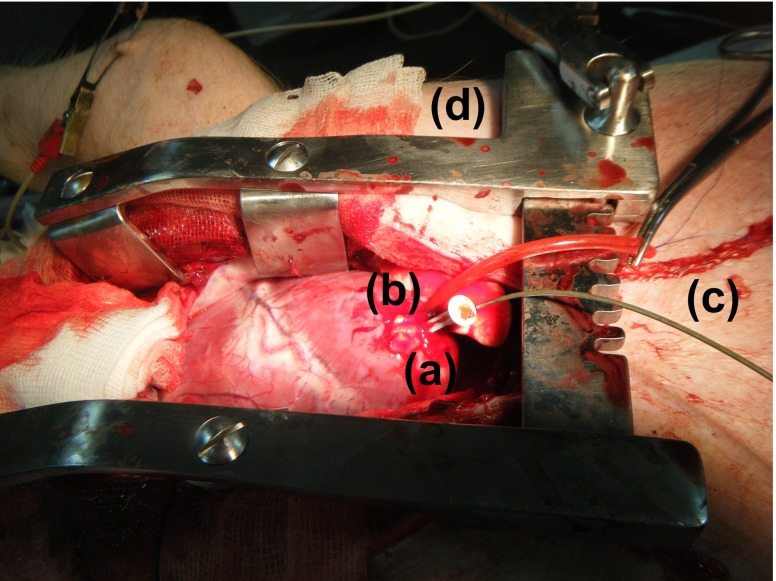
Anaesthetised mixed landrace pig with **a** exposed LV apex, **b** implanted wireless pressure sensor, **c** catheter-tip transducer and **d** chest spreader

Animal experiments were performed within the Elpen Pharmaceuticals Research Facility, Pikermi Attikis, Greece. A 30 kg mixed landrace pig was sedated and put under general anaesthesia (propofol, fentanyl, cisatracurium besylate). Sternotomy was performed to open the chest and expose the LV apex. A hole was opened in the apex and its opening was controlled by means of a purse string suture. The sensor was mounted on a plastic rod with a handle and inserted into the LV of the animal. The tip of a catheter-tip transducer, was inserted into the same hole and advanced into the cavity, again for the sake of comparison. Fig. 19 shows the setup for the experiment.

**Fig. 20:**
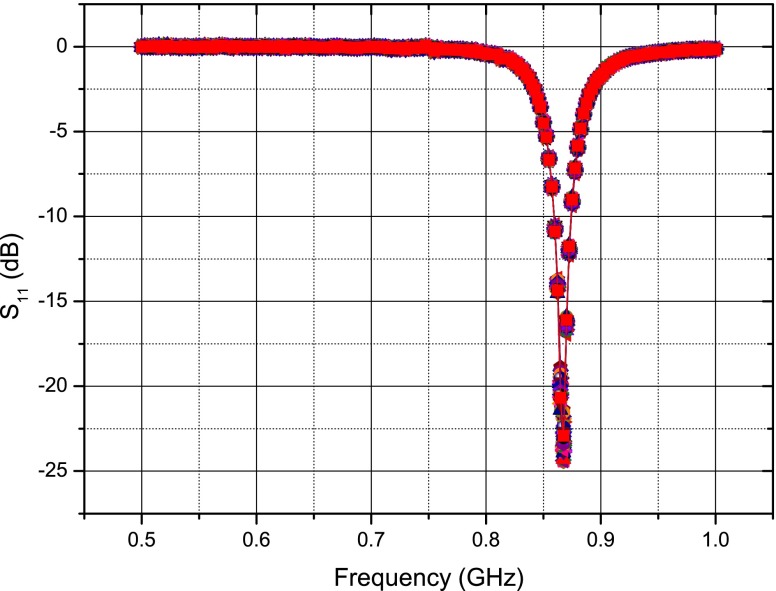
Stability of a patch antenna’s frequency response over time

**Fig. 21:**
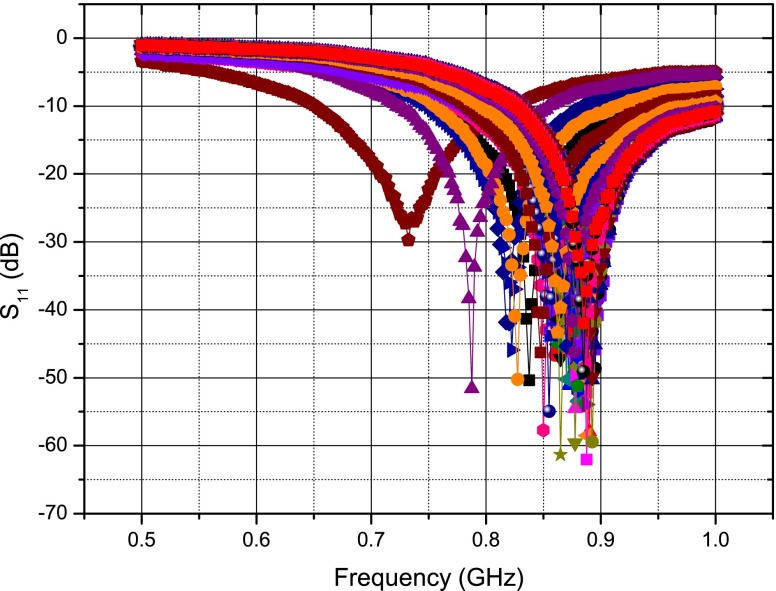
Instability of a dipole antenna’s frequency response over time

The implanted sensor was again activated by placing a tuned patch antenna close to the surface of the swine model’s chest. The patch is stable when placed close to the open chest and beating heart as seen in Fig. 20 where the reflection coefficient ($S_{11}$) of the patch is recorded 100 times over 8 s using a Rhode and Schwarz® ZVL Vector Network Analyzer. For comparison, the deflection of a dipole antenna’s frequency response versus time is shown in Fig. 21—showing significant frequency deviation, although some of this can be attributed to the large metal chest spreader used in the procedure, the dipole is clearly unsuitable as an external antenna.

**Fig. 22:**
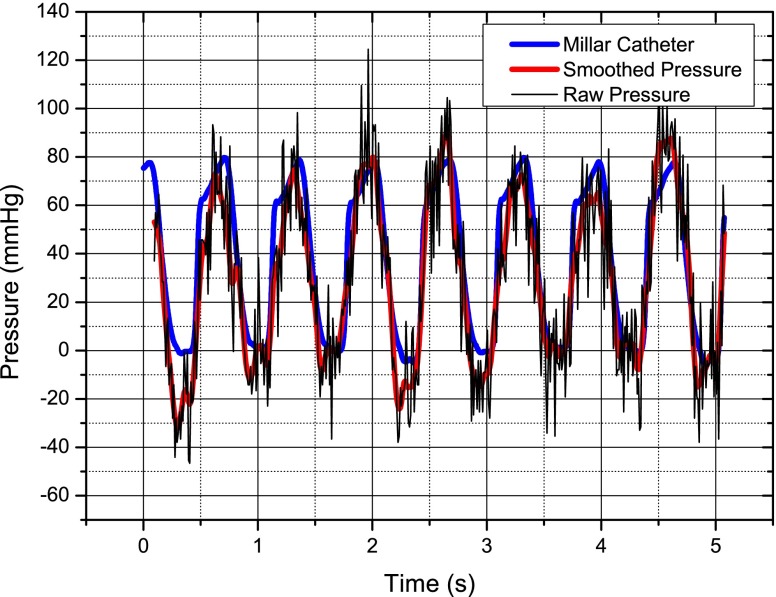
Left ventricle pressure response versus time

The same procedure is followed for the *in vivo* experiments as for the *in vitro* experiments to gather the frequency data. The baseline pressure is calibrated once the swine is euthanised. As for the *in vitro* experiment the gathered data from the wireless pressure sensor is smoothed and compares very well with that of the Millar catheter-tip transducer as seen in Fig. 22.

## Sensor limitations

It has to be pointed out that the described sensor was prototyped to demonstrate proof of concept and used as part of an implant to acquire wireless blood pressure data in an animal model. Due to acute nature of the study, the pressure sensor was only required to operate for a short time and therefore the choice of material as well as sensor construction method were not critical. The main problem with the current sensor is the lack of long time hermeticity of the sealed cavity. Polymers in general and silicone as well as PDMS in particular are known to be permeable to gases and certain liquids. Using polymers as a barrier results in gas exchange between the inside of the cavity and the blood stream over time and therefore will cause long term drifts in the sensor response. Quantification of the gas diffusion between the inner and the outer of the cavity is non-trivial: while air is richer in oxygen than blood, the opposite applies to carbon dioxide and the two exchange phenomena have opposite directions in the current configuration. Diffusivity of such gases through silicone membranes are of the order of 10^−5^ cm^2^/s (Robb 1968); relatively high if compared to an internal volume of 1.68 μl. Solutions for making the prototype suitable for long-term implantation are currently under study.

## Conclusion

A fully implantable pressure sensor system based on precise SAW resonators, which has the capability of providing continuous real-time wireless and therefore ambulatory blood pressure monitoring, has been presented in this paper. The results show that such a system compares very well with commercially available catheter-tip transducers. This system has the advantage of providing clinicians and surgeons with highly valuable and previously unavailable information regarding their patients. Future iterations of this system will address the current limitations of the sensor as well as a reduction in size of the sensor, antenna and interrogator with a view to assessing pressures in other cavities and vessels and leading to a fully certifiable system with a range of uses.
